# The iDiv Ecotron—A flexible research platform for multitrophic biodiversity research

**DOI:** 10.1002/ece3.8198

**Published:** 2021-10-04

**Authors:** Anja Schmidt, Jes Hines, Manfred Türke, François Buscot, Martin Schädler, Alexandra Weigelt, Alban Gebler, Stefan Klotz, Tao Liu, Sascha Reth, Stefan Trogisch, Jacques Roy, Christian Wirth, Nico Eisenhauer

**Affiliations:** ^1^ Helmholtz Centre for Environmental Research – UFZ Halle (Saale) Germany; ^2^ German Centre for Integrative Biodiversity Research (iDiv) Halle‐Jena‐Leipzig Leipzig Germany; ^3^ Leipzig University Leipzig Germany; ^4^ Key Laboratory of Vegetation Restoration and Management of Degraded Ecosystems South China Botanical Garden Chinese Academy of Sciences Guangzhou China; ^5^ Umwelt‐Geräte‐Technik GmbH – UGT Müncheberg Germany; ^6^ Martin Luther University Halle‐Wittenberg Halle (Saale) Germany; ^7^ French National Centre for Scientific Research – CNRS Paris France

**Keywords:** biodiversity and ecosystem functioning, biotic interactions, climate chambers, food webs, lysimeters, mesocosms

## Abstract

Across the globe, ecological communities are confronted with multiple global environmental change drivers, and they are responding in complex ways ranging from behavioral, physiological, and morphological changes within populations to changes in community composition and food web structure with consequences for ecosystem functioning. A better understanding of global change‐induced alterations of multitrophic biodiversity and the ecosystem‐level responses in terrestrial ecosystems requires holistic and integrative experimental approaches to manipulate and study complex communities and processes above and below the ground. We argue that mesocosm experiments fill a critical gap in this context, especially when based on ecological theory and coupled with microcosm experiments, field experiments, and observational studies of macroecological patterns. We describe the design and specifications of a novel terrestrial mesocosm facility, the iDiv Ecotron. It was developed to allow the setup and maintenance of complex communities and the manipulation of several abiotic factors in a near‐natural way, while simultaneously measuring multiple ecosystem functions. To demonstrate the capabilities of the facility, we provide a case study. This study shows that changes in aboveground multitrophic interactions caused by decreased predator densities can have cascading effects on the composition of belowground communities. The iDiv Ecotrons technical features, which allow for the assembly of an endless spectrum of ecosystem components, create the opportunity for collaboration among researchers with an equally broad spectrum of expertise. In the last part, we outline some of such components that will be implemented in future ecological experiments to be realized in the iDiv Ecotron.

## Graphical abstract

The iDiv Ecotron is a novel and unique research platform that was explicitly designed to address the perpetual claim that biodiversity–ecosystem function (BEF) research in terrestrial ecosystems needs to move beyond the manipulation of diversity at single trophic levels to embrace the multitrophic complexity of ecological communities.

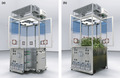

## 1 INTRODUCTION

Ecosystems are threatened by a multitude of environmental change drivers (Díaz et al., 2019; Maxwell et al., 2016; Murphy & Romanuk, 2014; Newbold et al., 2015; Pereira et al., 2012). Over the last few decades, there has been an explosion of studies examining changes in ecological communities and environmental conditions (Hines et al., 2019; Liu et al., 2011; Stork & Astrin, 2014). The desire to draw generalizable conclusions from these studies led to a period of synthesis, during which information from individual studies was compiled allowing for quantitative evaluation of the variation in ecological changes across systems (Gurevitch et al., 1992; Halpern et al., 2020; Hillebrand et al., 2020). Such comprehensive and quantitative synthesis studies enabled researchers to identify generalizable patterns in biodiversity (Calatayud et al., 2020), trends in biodiversity change (Blowes et al., 2019; Dornelas et al., 2014), and relationships between biodiversity and ecosystem functioning (e.g., Cardinale et al., 2012; Gessner et al., 2010; Lefcheck et al., 2015; Soliveres et al., 2016). These high‐impact synthesis studies can also serve as a roadmap for designing future experiments, as they help to identify important knowledge gaps which need to be filled in order to better understand the functioning of ecosystems and predict the consequences of climate change.

We have limited empirical evidence for at least three key aspects of environmental changes in ecosystems and communities that draw a roadmap for future research. First, there are limited numbers of ecosystem response variables that have been consistently studied across systems. For example, the most commonly reported response variables are primary production and decomposition (Cardinale et al., 2006; Schmidt, Auge, et al., 2015; Schmidt, John, et al., 2015). However, the few existing multitrophic biodiversity studies indicate that the interactions of higher trophic levels may be particularly important for multiple ecosystem functions (Hines, van der Putten, et al., 2015; Lefcheck et al., 2015; Naeem et al., 1994; Soliveres et al., 2016) and that especially these species might be very vulnerable to environmental changes (Hines, Eisenhauer, et al., 2015; Voigt et al., 2003). Second, studies tend to investigate limited types of mechanisms and processes underlying changes in biodiversity, ecosystem functioning, and the relationship between the two (Hillebrand et al., 2020). That is, while there is strong emphasis on the effects of global change drivers on changes in species richness (Tilman & Downing, 1994; Harpole et al., 2016, 1994; Seabloom et al., 2021, but see Dornelas et al., 2014; Vellend et al., 2013), there is less known about the ecosystem consequences of changes in behavior (Cordero‐Rivera, 2017; Wilson et al., 2020) and community composition (Hillebrand et al., 2018; Spaak et al., 2017) of species that persist in communities. Third, although ecosystems are confronted with complex cocktails of global change drivers (Bowler et al., 2020), so far only a limited number of their types and combinations have been studied in realistic experiments (Rineau et al., 2019; Rillig et al., 2019, but see Schädler et al., 2019; Korell et al., 2020). Especially with regard to climate change, understanding interactions between different environmental variables such as temperature and precipitation, land use or biodiversity on ecosystem functioning is essential to make predictions for future ecosystem developments and the potential consequences for society (Roy et al., 2017). To address our current knowledge gaps, we need experiments which can simultaneously manipulate and measure different global change drivers (Vanderkelen et al., 2020) and investigate their impacts on a wide range of functional groups and trophic levels of organisms (De Boeck et al., 2020; Komatsu et al., 2019; Korell et al., 2020). Combining such “meta‐scale” studies with laboratory and field studies, especially large‐scale climate change experiments (like Schädler et al., 2019), provides the opportunity to understand the complex patterns of biodiversity–ecosystem function relationships and their responses to environmental changes as well as the underlying processes that operate across organizational levels of life (cell‐individual‐population‐community‐ecosystem; Ferlian et al., 2018).

Here, we introduce the iDiv Ecotron platform (iDiv stands for the German Centre for Integrative Biodiversity Research Halle‐Jena‐Leipzig in Germany). This platform is a highly flexible experimental infrastructure that was specifically designed to perform multitrophic biodiversity experiments in terrestrial ecosystems (Eisenhauer & Türke, 2018). In the following sections, we describe the iDiv Ecotron specifications and functioning, we highlight a case study experiment as an application possibility, and we provide an outlook on the potential contributions of future ecotron experiments. The concept of the iDiv Ecotron was to create a facility which allows the setup and maintenance of complex communities and manipulation of several abiotic factors in a near‐natural way, while simultaneously measuring multiple ecosystem functions. Environmental conditions, such as humidity, nutrient supply, light, and precipitation, can be fully controlled and monitored (for details see Appendix 1), which allows the iDiv Ecotron to be used for the simulation of multiple abiotic scenarios together with scenarios of above‐belowground community change. The iDiv Ecotron offers the possibility to study a wide range of ecosystem responses, including above‐belowground interactions of plants, microbes, and invertebrates. The platform can accommodate stand‐alone experiments and also provides complementary information to small‐ and large‐scale experiments (lab‐ecotron‐field). Therefore, the iDiv Ecotron links investigations at multiple experimental and spatial scales and serves as a key component for collaborations between researchers from different disciplines to conduct interdisciplinary studies on the drivers of, and relationship between, biodiversity and ecosystem functioning. Consequently, this platform is likely to provide novel insights into ecosystem responses to global change.

## 2 SETUP AND DESIGN OF THE iDiv ECOTRON

Based on some first facilities that were built in Germany (ExpoSCREEN Munich, Payer et al., 1987), England (Imperial College ecotron in Silwood Park; Lawton, 1996; Lawton et al., 1993) and the United States (Desert Institute EcoCELLs in Reno, Nevada, Griffin et al., 1996) in the 1980s and 1990s, highly sophisticated experimental infrastructures, so‐called “ecotrons,” started to get established worldwide in the early century, reflecting the urgent need for such infrastructures accompanied by the rapid evolution in digital technology and electronics (e.g., Ecotron in Montpellier, France, Milcu et al., 2014; IleDeFrance Ecotron EcoLabs in Saint‐Pierre‐lès‐Nemours, France, Verdier et al., 2014; Ecotron in Hasselt, Belgium; Biotron in Lincoln, New Zealand). These types of facilities started to go beyond single trophic levels (mainly plants), like the so‐called “phytotrons” that were emerging in the 1950s and 1960s (e.g., the Duke University, https://biology.duke.edu/facilities/phytotron; or the North Carolina State, https://phytotron.ncsu.edu/; see Roy et al., 2021 “supinfo‐0003”). The idea behind an ecotron is to combine the precision, specificity, and complete control of single independent and response variables of laboratory experiments and the realism and large‐scale community‐ and environment‐related aspects of field studies. Roy et al. (2021) define an ecotron as an “…experimental facility comprising a set of replicated enclosures designed to host ecosystems samples, enabling realistic simulation of above‐ and belowground environmental conditions, while simultaneously and automatically measuring ecosystem processes. Therefore, ecotrons provide continuous information on ecosystem functioning (fluxes of energy and matter).” The Silwood Park Ecotron in particular has focused research on multitrophic interactions (see Lawton, 1996; Lawton et al., 1993). The iDiv Ecotron continues the tradition of aboveground–belowground work by creating a facility capable of housing a multitude of above‐ and belowground organisms from various trophic groups in a large number of single independent chambers (unlike other indoor facilities, such as ExpoSCREEN in Munich, Germany, or the Montpellier Ecotron mesocosms in France; see Roy et al., 2021) while being completely independent from external weather conditions (unlike, for example, the Hasselt Ecotron in Belgium).

**FIGURE 1:**
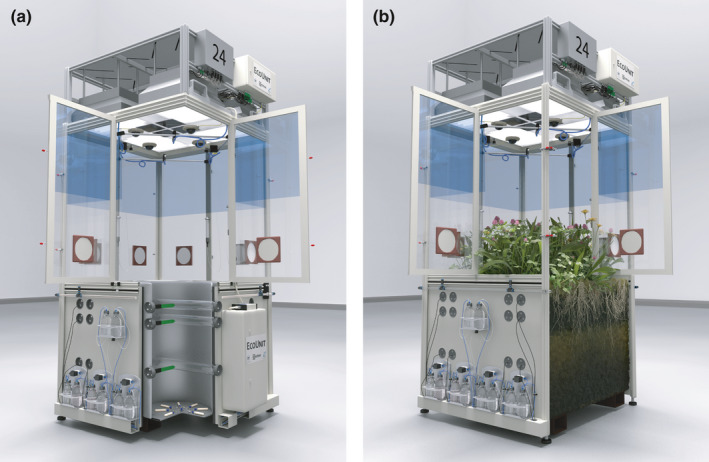
Illustration of an EcoUnit; (a) construction drawing with corner cutout to visualize the technical interior features; (b) EcoUnit with earth‐filled lower part, upper part equipped with illustrative vegetation

A review with detailed descriptions and comparisons of a variety of current ecotrons worldwide can be found in Roy et al. (2021). However, the breadth of ecotrons compared in Roy et al. (2021) prevents an in‐depth examination of any one facility. With the goal of inspiring collaborative proposals to use the research platform, and to provide a reference for the design and specification of the facility for future research, we provide an in‐depth description of the iDiv Ecotron here. The iDiv Ecotron is located in a climate‐controlled and blacked out hall on an area of 485 m^2^ at the research station of the Helmholtz Centre for Environmental Research—UFZ in Bad Lauchstädt (Saxony‐Anhalt, 51°22′60N, 11°50′60E, 118 m a.s.l.), Germany. The indoor research facility houses 24 identical experimental units (hereafter EcoUnits, see Figure 1), each of which can contain one to four ecosystems, separated above‐ or belowground, or both. In this way, up to 96 subunits with various biotic and abiotic variables to be manipulated and measured independently can be set up. The iDiv Ecotron concept was developed in cooperation with numerous scientists and technicians from iDiv, including strong participation by the UFZ, national and international collaborators, and the companies “EMC – Gesellschaft zur Erfassung und Bewertung von Umweltdaten mbH,” and “Umwelt‐Geräte‐Technik GmbH (UGT), Müncheberg.”

EcoUnits are experimental chambers with the outer dimensions of 1.55 m × 1.55 m × 3.20 m (*L* × *W* × *H*), comprising a lower part, which can be filled with soil (belowground part), an upper part (aboveground part), and a technical section on the top. The frame of the chamber is constructed of aluminum construction profiles providing stability and flexibility.

The belowground part contains a container with internal dimensions of 1.24 m × 1.24 m × 0.80 m (*L* × *W* × *H*) made of welded PE‐HD and a steel bottom. It can be filled with up to 1.23 m^3^ of soil, or alternatively equipped with four steel cylinders (lysimeters) measuring 0.50 m × 0.80 m (*D* × *H*), each of which can hold 0.16 m^3^ of soil. The container as well as the lysimeters feature pluggable openings in three different depths (9.5, 21.5, and 43.5 cm), where sensors for soil temperature, soil moisture, and water potential can be inserted. Additional larger openings in the same depths as those for the sensors offer the opportunity to install minirhizotrons (acrylic glass tubes) for horizontal monitoring of root development using a portable root scanner (see Möller et al., 2019).

Besides manually filling the lysimeters with soil, they can be used to excavate intact soil monoliths, including aboveground vegetation, directly from the field. This enables precise investigations of almost undisturbed soil systems, preserving their structure and stratification as well as their faunal and microbial soil communities. Both the lysimeters and the containers provide a living space of sufficient size to establish and study belowground organisms and processes. To achieve a near‐natural soil temperature gradient with temperature decreasing from the surface to deeper soil depths, the bottom of the soil container was fitted with a coil that circulates a cooling medium. This system can be regulated individually for each EcoUnit and automated with the data from the above‐ and belowground temperature sensors.

To allow pore water sampling and near‐natural drainage of water from the soil system, four suction systems are installed at the bottom of the soil container or one in each lysimeter. Each suction system consists of a suction cup ring with 8 suction cups, a pump, a control module, and two glass bottles. By applying negative pressure (max. −60 kPa), the suction systems continuously extract and collect pore water. When one bottle is filled, the control unit of each suction system automatically switches to the alternate bottle and empties the first one. To quantify the volume of water sampled, the system counts the number of bottle changes. This enables a continuous supply of soil water for chemical analyses and an automated recording of the total amount of collected water. Simultaneously, the negative pressure applied at the bottom of the lysimeter lowers the water potential from there up and reduces “unnatural” high plant transpiration. When the soil column is cut over the course of the monolith extraction, the water potential at the cut level becomes zero—it is brought to atmospheric pressure, which eases and therefore increases the extraction of water by plants. Here, the suction system can be used to apply the pressure that corresponds to the natural in situ water potential at that depth. This allows for these ecosystems to further approximate natural conditions (Groh et al., 2016). Optionally, single suction cups can also be installed in three different depths (9.5, 21.5, and 43.5 cm) by using the pluggable openings.

**FIGURE 2:**
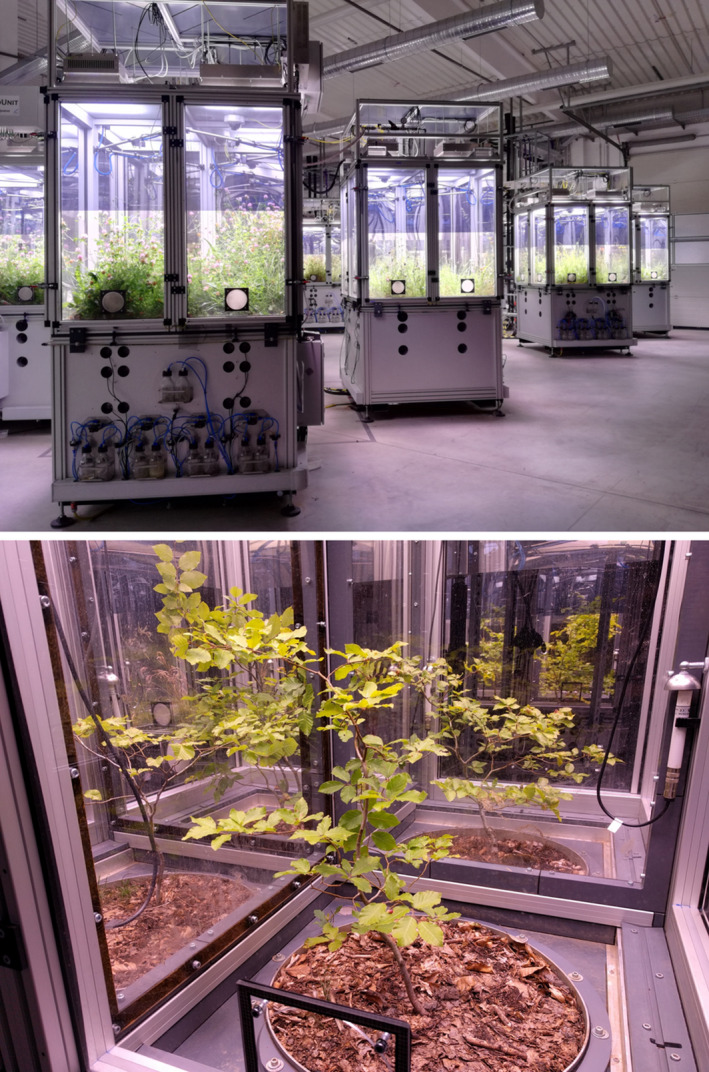
Grassland (upper picture) and tree saplings (bottom picture) planted in EcoUnits of the iDiv Ecotron

The aboveground part, with internal dimensions of 1.46 m × 1.46 m × 1.50 m (*L* × *W* × *H*), provides sufficient space for communities of large herbs or tree saplings (see Figure 2) including their complex multitrophic interaction networks. In each quarter, a video camera can be installed (for details on the camera system, see Appendix 1), for example, for monitoring vegetation development over time (Ulrich et al., 2020) or insect behavior, such as movement patterns, flower visitation of pollinators, and habitat use. By using infrared lights, the cameras can also operate in darkness.

The aboveground part is further equipped with an irrigation system consisting of a flow meter and four electromagnetic valves with fixed nozzles. By sequentially processing the opening times of the valves, each quarter of an EcoUnit can be automatically provided with individual volumes of water at programmable times. All irrigation systems are supplied with deionized water from a central reverse osmosis system. To compensate for the flow resistance caused by different lengths of supply hoses to each EcoUnit, the water pressure at the water treatment plant is increased to approx. 4 bar (400 kPa) and then reduced to a constant level of about 2 bar (200 kPa).

Ambient air temperature is maintained centrally in the Ecotron hall, but the air flow rate of each subunit can be regulated individually. Climatic conditions are recorded by combined humidity and temperature sensors installed in each quarter of an EcoUnit, usually placed at a height of 40 cm above soil surface. Conditions are continuously compared with those of the hall and, as needed, automatically adjusted by increasing or decreasing the fan speed of the ventilation system. All four quarters of the EcoUnit can be regulated individually.

Further, the top part of the EcoUnits is equipped with a diffuser holding 4 LED lamps adjustable in color and intensity. The light system provides three individually dimmable color channels (400–405, 460–475, 625–720 nm) as well as a dimmable white channel (5000 K + 3000 K), and a binary (ON/OFF) infrared channel (840–850 nm). For the overall luminance as well as for each color channel, the intensity can be set from 0% to 100% individually, determining the general light color. This can be done either manually or automated in an hourly resolution with an automatically linear transition between the settings. In this way, the relative proportion of different wavelengths within the light spectrum can be modified (e.g., a higher proportion of red light at dawn and dusk). The maximum photosynthetic active radiation (PAR) 5 cm above the standard soil surface can reach about 400 μmol s^−1^ m^−2^ on average (detailed information on the heterogeneity of illumination can be found in Appendix 2). Two electrical cabinets provide the power supply for the lamps and a local control unit for all sensors and actuators.

Control commands and settings of all manipulable environmental parameters are stored in a central database and get transmitted to each EcoUnit via a network. In turn, the execution confirmations as well as the timestamped sensor data of each EcoUnit are logged in the same database. This asynchronous communication between EcoUnits and database server provides a high operational reliability and independence of network's capacity bottlenecks. A simple graphical user interface eases the handling of database entries.

## 3 CASE STUDY–EFFECTS OF ABOVEGROUND PREDATORS ON ABOVEGROUND–BELOWGROUND INTERACTIONS AND ECOSYSTEM FUNCTIONS

### 3.1 Rationale

Aboveground–belowground interactions are known to determine the functioning of terrestrial ecosystems (Scheu, 2001; Wardle et al., 2004). Previous work has shown that aboveground invertebrate predators can induce trophic cascades that “trickle‐down” to affect soil food webs and a broad range of ecosystem functions (Wardle et al., 2005). Here, we present a case study conducted in the iDiv Ecotron to test how plant community composition may affect such trickle‐down effects. Further, as plant‐mediated effects of aboveground predators may additionally depend on the activity of soil ecosystem engineers, which structure the environment for (Brown, 1995; Eisenhauer, 2010) and the resource supply of soil food webs (Eisenhauer, 2010; Schwarzmuller et al., 2015), we investigated the effects of soil fauna on multitrophic diversity and ecosystem functions. The unique functionality of the iDiv Ecotron enabled us to study potential cascading effects of aboveground predators on herbivores, plants, and soil food webs, and how these effects are modulated by decomposer communities in the soil. Specifically, we tested (1) if the target plant biomass would be lower in the presence of herbivores, an effect that would be alleviated by the presence and higher density of predators (e.g., Wardle et al., 2005). We further hypothesized (2) that the identity of the neighboring plant community will affect the biomass of the target plant with biomass being higher in a community with herb species compared to grass species due to elevated competition for soil resources in the presence of grasses (Eisenhauer & Scheu, 2008). Moreover, we expected (3) the presence of decomposers (earthworms and Collembola) to affect the tritrophic interactions aboveground, as decomposition and mineralization processes in soil can significantly alter the performance of the target plant (van Groenigen et al., 2014; Scheu, 2003) as well as the competition with the surrounding vegetation (Eisenhauer & Scheu, 2008; Sabais et al., 2012). Finally, we hypothesized (4) that there will be trickle‐down effects of aboveground predators on soil nematode density and species richness due to altered resource supply and that soil food web responses to these trickle‐down effects will be modulated by earthworm presence as they significantly change the structure of the environment for and resource supply of other soil organisms (Brown, 1995; Eisenhauer, 2010).

### 3.2 Methods

#### Experimental setup and data analyses

**FIGURE 3:**
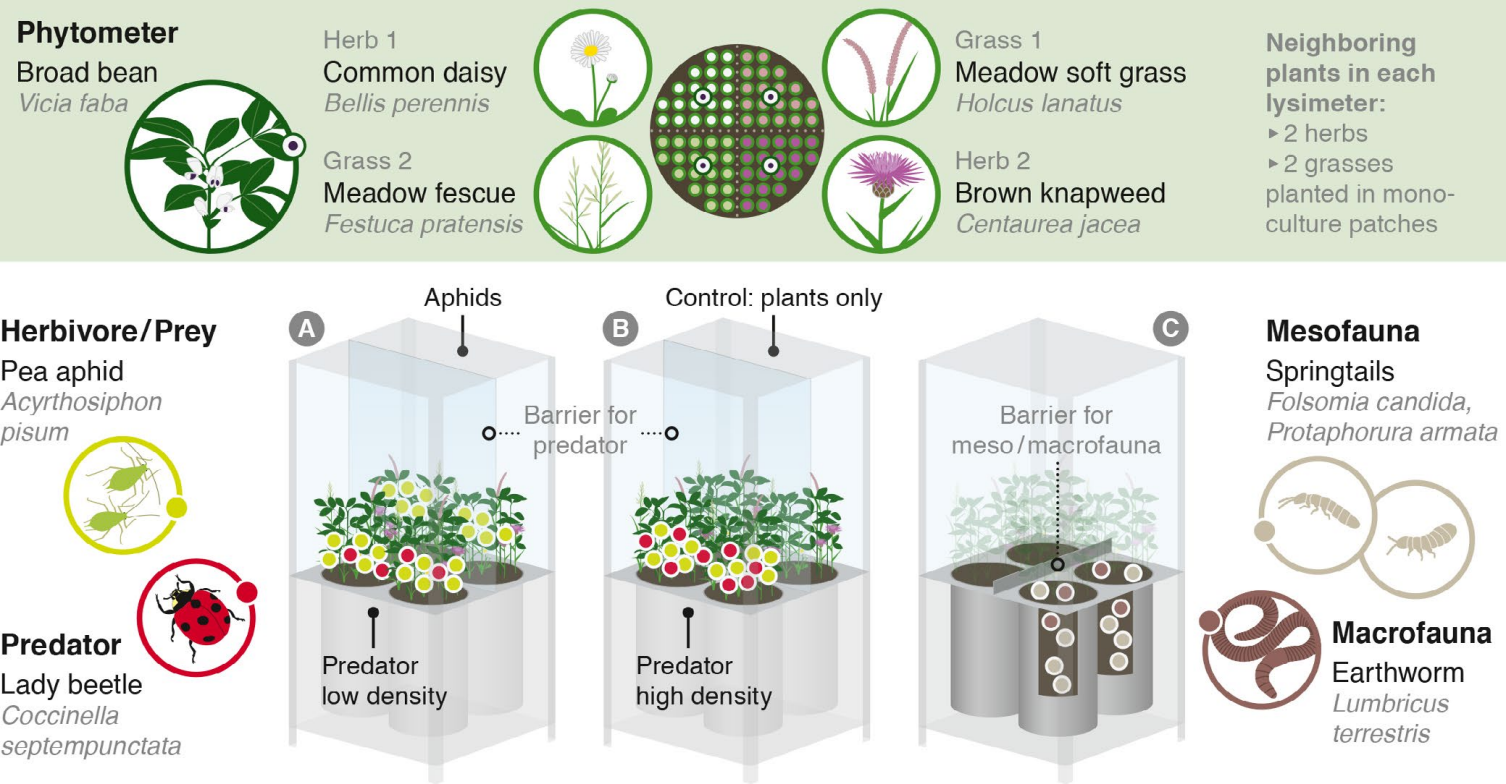
Experimental setup of the case study

In six EcoUnits in a lysimeter configuration, a tritrophic system got established comprising a target plant (*Vicia faba* L.), its host‐specific aphid (*Acyrthosiphon pisum* Harris), and a predator exclusively feeding on aphids (*Coccinella septempunctata* Linnaeus; details on initial densities can be found in Appendix 3). We further included a soil fauna treatment (with and without soil fauna) to test whether predator effects are modulated by the presence of macro‐ and meso‐decomposers in the soil; and a “plant neighbor” treatment to test plant responses in different competitive environments and to increase variation for reproducibility purposes (Milcu et al., 2018). Concisely, we established an experimental setup with three treatment factors comprising *aboveground invertebrates*, *belowground invertebrates*, and *surrounding vegetation* (see Figure 3). Each treatment combination was replicated three times. While soil compartments were all fully isolated one from another (four per EcoUnit), the aboveground compartments allowed for an exchange of invertebrates between lysimeter pairs with an acrylic glass barrier of 15 cm height preventing the migration of soil invertebrates between lysimeters. In this way, there were two independent experimental units in each of the six EcoUnits resulting in twelve independent “Sub‐units” and 24 “Sub‐sub‐units” in total (more details on the experimental setup and environmental conditions can be found in Appendix 4). The experiment ran for 124 days, from February 03, 2017, to June 06, 2017.

A general linear mixed model (GLMM) type III sum of squares (procedure MIXED, SAS 9.2) was used to analyze dry weight (g) of the focal plant (*Vicia faba*), nematode density, nematode species richness (all three recorded during the harvest at the end of the experiment), maximum numbers of aphids (peak number of individuals counted in one assessment during the experiment), and days of aphid infestation (number of days beans were infested with aphids; details can be found in Appendix 5) in relation to the fixed factors *aboveground invertebrates*, *belowground invertebrates*, and *surrounding vegetation*. The factor “Sub‐unit” nested in “Sub‐sub‐unit” was considered random. Post hoc Tukey's HSD tests were carried out to reveal significant differences between the respective factor levels within factors.

##### Details on treatment factors:

1. *Aboveground invertebrates*: The treatment was established to test whether predator effects depend on their density (*4* *levels*: all aboveground invertebrates absent *[Control]*, only aboveground herbivores present *[Herbivores only]*, aboveground herbivores present with aboveground predators in low density *[Coccinella low]*, aboveground herbivores present with aboveground predators in high density *[Coccinella high]*).
2. *Belowground invertebrates*: To half of the lysimeters earthworms and Collembola were added to test if predator performance is modulated by the presence of macro‐ and meso‐decomposers in the soil (*2* *levels*: earthworms and Collembola present *[with soil fauna]* versus earthworms and Collembola absent *[no soil fauna]*); soil invertebrate species list and initial densities can be found in Appendix 5).
3. *Surrounding vegetation*: the focal plants (*Vicia faba* L) were each surrounded by a herb or grass monoculture (*4* *levels*: *Bellis perennis* L., *Centaurea jacea* L., *Festuca pratensis* Huds., *Holcus lanatus* L.; details on plants can be found in Appendix 7).

### 3.3 Results

**FIGURE 4:**
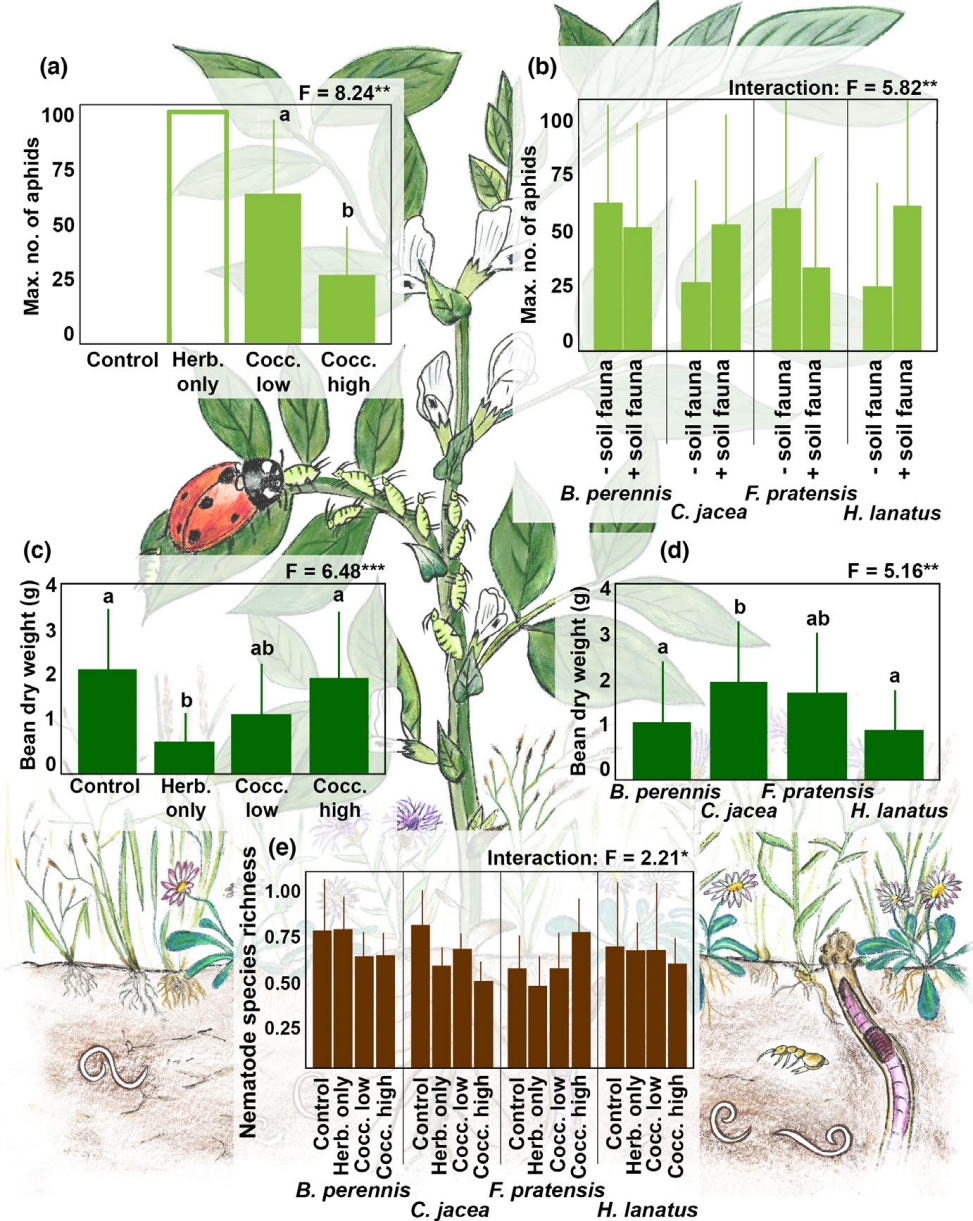
Effects of (a) aboveground invertebrate treatment (control, herbivores only, *Coccinella* low, *Coccinella* high; details in Appendix 3) as well as (b) the interaction of belowground invertebrate presence (with/+ soil fauna, without/‐ soil fauna) and bean plant neighbor species identity (*Bellis perennis* L., *Centaurea jacea* L., *Festuca pratensis* Huds., *Holcus lanatus* L.) on the maximum number of aphids; (c) aboveground invertebrate treatment and (d) bean plant neighbor species identity on bean dry weight; and (e) the interaction of aboveground invertebrate treatment and bean plant neighbor species identity on the species richness of nematodes. *p = .05; **p < .01; ***p < .001. For detailed results see Table A3

The target plant (for brevity “bean” in the following) dry weight differed significantly depending on the neighboring plant species (*F*_3,48_ = 5.16, *p* < .01; Figure 4, Table A3) and the aboveground invertebrate treatments (*F*_3,48_ = 6.48, *p* < .001; Figure 4, Table A3), whereas it did not differ among belowground invertebrate treatments as well as with any of the two‐ or three‐way interactions of the three variables tested. Bean dry weight was lowest in patches with *B*. *perennis* and *H*. *lanatus*, whereas it was significantly higher in *C*. *jacea* patches (Figure 4). Furthermore, bean dry weight was highest in the aboveground invertebrate “*Control”* and the “*Coccinella high”* treatments, whereas it was lowest in the “*Herbivores only”* treatment.

The maximum number of aphids and number of days of aphid infestation differed significantly between the aboveground invertebrate treatments (*F*_1,24_ = 8.24, *p* = .01; Figure 4, Table A3; and *F*_3,48_ = 63.19, *p* < .001, respectively; Table A3). Further, the maximum number of aphids showed significant differences in the interaction between plant neighbor species and belowground invertebrates (*F*_3,24_ = 5.82, *p* = <.01; Figure 4, Table A3). In general, numbers of aphids were higher in the “*Coccinella low”* treatment compared to the “*Coccinella high”* treatment. Depending on the plant neighbor identity, maximum number of aphids slightly decreased (*B*. *perennis* and *F*. *pratensis*) or increased (*C*. *jacea* and *H*. *lanatus*) with the presence of belowground invertebrates, but effects were not statistically significant.

Nematode densities differed significantly only between plant neighbor species (*F*_3,48_ = 2.86, *p* = .05; Table A3). Highest numbers were found in patches where *C*. *jacea* was planted and lowest numbers in plots with *F*. *pratensis* (significant differences were found only between these two). For nematode species richness, only the interaction between plant neighbor species and the aboveground invertebrate treatment was significant (*F*_9,48_ = 2.21, *p* = .04; Figure 4, Table A3). Although the post hoc Tukey's HSD test showed no significant differences between factor levels, nematode species richness was lowest in the “*Herbivores only”* treatment in the presence of *F*. *pratensis*, while it was highest in the “Control” treatment in the presence of *C*. *jacea*.

### 3.4 Discussion

In contrast to our expectations, beans did not generally benefit from growing in herb communities, while being suppressed by more dominant nitrophilous grasses (Eisenhauer & Scheu, 2008). We observed opposing effects for the two grass species and for the two herb species on bean biomass. Among the four neighboring plant species, *H*. *lanatus* produced by far the highest amount of aboveground plant biomass (139.5 g) at the end of the experiment compared to the other three species (*F*. *pratensis*: 92.1 g, *C*. *jacea*: 51.1 g, *B*. *perennis*: 5.3 g), and, as graminoid species typically produce a dense and large root system, we speculate that also root biomass was highest (not assessed in this study). Thus, both enhanced aboveground light competition and belowground competition for resources may have contributed to an overall advantage in resource acquisition over the bean, causing low bean biomass. Indeed, it has been often confirmed that grasses are stronger competitors compared to herbaceous species (Del‐Val & Crawley, 2005; Tilman, 1982). Moreover, another potential explanation for the patterns found in our study may be that in patches of low biomass, for example, in *B*. *perennis* patches, the habitat structure for predators was comparably low leading to a migration to more favorable habitat structures. This effect may have cascaded to lower trophic levels increasing abundances of herbivores and decreasing plant performance (Romero & Koricheva, 2011). The importance of such non‐trophic interactions based on habitat structure has been often highlighted (Kalinkat et al., 2013; Majdi et al., 2014).

Our results confirm the often found tritrophic relationships between predators, herbivores, and primary producers, where predators, in our case ladybirds, exert a top‐down control on aphid abundances which, in turn, have a top‐down effect on the bean (Romero & Koricheva, 2011). Surprisingly, the effects of plant neighbor species on aphid abundances were opposing for communities without and with belowground invertebrates. These findings highlight the significance of aboveground–belowground interactions and show that decomposers can influence aboveground multitrophic interactions by altering the competition between plants (Wardle et al., 2004). Moreover, we found that trickle‐down effects of aboveground invertebrates on soil food webs (here represented by soil nematode species richness) depend on plant community composition. This finding suggests that the competitive environment of a focal plant can alter its effects on soil community composition, potentially through changes in the amount and quality of plant‐derived resources entering the soil (Hooper et al., 2000).

Taken together, our study shows distinct interaction effects between aboveground and belowground invertebrate communities on multitrophic interactions and community composition in the sub‐compartments. These changes are likely to alter how communities function, which may have subsequent feedback effects on nutrient cycling and community composition. The results of our study highlight the need for infrastructures that allow to manipulate food webs of high complexity, which can hardly be realized experimentally under field or simplified laboratory conditions (Beyers & Odum, 1993), and at the same time, taking advantage of measuring and controlling a large fraction of other non‐targeted parameters including environmental conditions.

## 4 OUTLOOK

Over the last several decades, ecologists have written thousands of papers about changes in climate and biological communities. Yet, some important knowledge gaps remain. Here, we discuss the relevance of mesocosm research as an underappreciated scale of inquiry. The utility of mesocosm/Ecotron experiments is not limited to terrestrial systems, and similar rationale has been used to promote independent aquatic mesocosm facilities (e.g., Hines et al., 2013), as well as consortia of aquatic facilities (e.g., Mesoaqua, https://cordis.europa.eu/project/id/228224/reporting; Aquacosm, https://www.aquacosm.eu/project‐information/). However, we focus on terrestrial systems here, because we further develop this line of reasoning by describing three opportunities where the iDiv Ecotron is particularly well suited to address challenges limiting an integrative understanding of biodiversity and ecosystem functioning.

Mesoecology is an important and often overlooked scale in environmental change research (Stewart et al., 2013). While macroecological studies provide more realistic abiotic and biotic context for investigating ecosystem processes, complex communities and environmental conditions can only be controlled, and causality of patterns inferred, to a very limited extent, and often with very few replicates (Eisenhauer & Türke, 2018; Lawton et al., 1993). On the other hand, laboratory microcosm studies can fully control and alter external factors and allow for high replication (Benton et al., 2007). However, laboratory studies are often limited to investigating single mechanisms and processes under artificial and simplified environmental conditions (Lawton et al., 1993). They are prone to experimental artifacts caused by the simplification of complex interactions which may bias results and induce misleading conclusions (Carpenter, 1996, 1999; Milcu et al., 2018; Roy et al., 2021; Schindler, 1998). The iDiv Ecotron provides an important middle ground, especially with the possibility of extracting and implementing up to 96 intact soil monoliths which allows for precise investigations of almost undisturbed soil systems, while preserving their structure and stratification as well as their faunal and microbial soil communities. Mesocosm experiments close the gap between small‐ and large‐scale studies and they allow scientists working together across levels of organization from cells to ecosystems to test basic and applied ecological questions. However, attempts to do so will profit from including a few key aspects of research that serve as future opportunities.

### Opportunity 1: Multitrophic diversity change

Although many studies have evaluated responses of plant species to environmental variation, ecologists have yet to demonstrate the collective importance of these responses for the full complement of plants’ interaction partners above and below the ground. This is particularly important because not all taxa that interact with plants perceive environmental variation at the same scale (Heinen et al., 2018; Veen et al., 2019). Therefore, although it has been shown that diversity can beget diversity, and patterns in plant diversity can parallel patterns of soil diversity and aboveground consumer diversity (Eisenhauer et al., 2013; Scherber et al., 2010), these patterns may be mismatched (Cameron et al., 2019) and/or further decoupled by environmental change drivers (Bardgett & Wardle, 2010; Thakur, 2020). Future iDiv Ecotron experiments will evaluate differences in spatial and temporal response to drivers that may explain mismatches in above‐ and belowground biodiversity (Eisenhauer & Türke, 2018). The iDiv Ecotron allows for simultaneous manipulation of aboveground and belowground biodiversity, with particular emphasis on belowground sub‐systems through the use of intact soil cores, the examination of roots via rhizotrons, and large enough spatial scale to examine differences in patterns of aboveground and belowground diversity. Rigorously testing factors that influence aboveground–belowground relationships is critical, because they form key pathways by which environmental variation influences community assembly, biodiversity effects on ecosystem functioning, and the impacts of environmental change on community dynamics. To develop effective plans to conserve biodiversity, we need meso‐scale empirical studies that test the mechanisms underlying effects of environmental drivers on aboveground–belowground biodiversity and ecosystem functioning.

### Opportunity 2: Beyond presence/absence—Behavioral and chemical mechanisms of plants and animal interactions

Traditionally, experimental examinations of food web interactions have been conducted by stocking simplified communities into microcosms or field plots and quantifying the outcome of the interactions by counting the presence and abundance of species after a designated time period. It is likely that phenotypic changes (e.g., changes in behavior, chemistry, or morphology) serve as precursors to the numerical changes in community composition that are typically quantified, or that phenotypic changes can drive major changes in ecosystem functioning on their own (Matthews et al., 2011; Turcotte & Levine, 2016). Yet, phenotypic responses are more often evaluated in highly simplified communities with limited emphasis on interaction complexity. We see considerable potential for iDiv Ecotron studies to extend highly simplified laboratory experiments showing effects of environmental drivers on phenotypic responses (e.g., behavioral, morphological, and physiological change). Changes in local foraging and behavior/activity patterns may be an important mechanism underlying changes in biodiversity–ecosystem function relationships (Jeltsch et al., 2013). The iDiv Ecotron can be fit with a landscape of sensors for detecting movement of animals tagged with RFID chips. Repulsed (or aggregated) animal activity patterns can point to the importance of non‐trophic and trait‐mediated interactions (e.g., fear). Such behavioral changes are not limited to animals. For example, behavior changes of plants emission of plant volatiles can be turned off and on depending on plant interaction partners. Plant volatiles play key roles in plant defense against aboveground and belowground herbivores, plant competition, and plant communication (Pierik et al., 2014). Yet, research of plant volatiles is often conducted on isolated plants or pairs of plants. These aspects of phenotypic changes (animal movement, plant volatiles) are difficult to assess in field conditions where signals may be detected by ecological communities but not my scientific instruments due to difficulties relocating animals in larger more complex landscapes, or buffering effects of wind. Future iDiv Ecotron experiments will examine the role of aboveground–belowground plant and animal behavior in complex communities.

### Opportunity 3: Multiple drivers of environmental heterogeneity and environmental change

We have only begun to identify the full array of environmental changes confronting ecosystems today (Bowler et al., 2020). The iDiv Ecotron allows for independent manipulation of several abiotic factors (e.g. precipitation, light, nutrients, and temperature) in gradient‐based or factorial combinations. Non‐additive, synergistic, or unexpected responses may be detected from heretofore untested combinations of environmental change drivers. There is also much potential to use the iDiv Ecotron to examine the influence of minor or extreme levels of drivers and to detect non‐linear relationships between drivers and ecosystem responses (De Boeck et al., 2015; Damgaard et al., 2018). Therefore, the iDiv Ecotron is an ideal tool to complement environmental change experiments where ecological responses are evaluated over longer time periods or greater spatial scales, but at the cost of examining a reduced number of scenarios (e.g., Schädler et al., 2019). Future studies may therefore be considered as a step toward precision and mechanistic understanding supplementing other laboratory or field studies.

In conclusion, the iDiv Ecotron provides a flexible collaborative research platform that operates at an intermediate scale, connecting simplistic microcosm experiments and real‐world heterogeneity. Their size allows for evaluation of naturally complex aboveground–belowground interactions, often overlooked mechanisms (e.g., behavior, plant volatiles), as well as a broad range of environmental drivers. Therefore, this robust experimental facility can help to fill several critical knowledge gaps identified in synthesis studies. The iDiv Ecotron will be used to assemble, disassemble, and reassemble ecological communities in rigorous tests of basic and applied ecological questions. We start with an empty box with strong technical capabilities to control environmental conditions, endless possible combinations of species, and an open call to potential collaborators: What would you do if you could rebuild the world?

## CONFLICT OF INTEREST

The authors declare no conflicts of interests.

## AUTHOR CONTRIBUTIONS

**Anja Schmidt:** Data curation (lead); Formal analysis (lead); Investigation (equal); Visualization (lead); Writing‐original draft (lead); Writing‐review & editing (lead). **Jes Hines:** Validation (equal); Writing‐original draft (equal); Writing‐review & editing (equal). **Manfred Türke:** Conceptualization (equal); Data curation (lead); Investigation (equal); Methodology (lead); Project administration (lead); Supervision (lead); Writing‐review & editing (equal). **François Buscot:** Conceptualization (equal); Funding acquisition (equal); Investigation (equal); Validation (equal); Writing‐review & editing (equal). **Martin Schädler:** Conceptualization (equal); Funding acquisition (equal); Investigation (equal); Validation (equal); Writing‐review & editing (equal). **Alexandra Weigelt:** Conceptualization (equal); Funding acquisition (equal); Investigation (equal); Validation (equal); Writing‐review & editing (equal). **Alban Gebler:** Conceptualization (equal); Investigation (equal); Software (equal); Writing‐review & editing (equal). **Stefan Klotz:** Conceptualization (equal); Methodology (equal); Writing‐review & editing (equal). **Tao Liu:** Investigation (equal); Methodology (equal); Writing‐review & editing (equal). **Sascha Reth:** Conceptualization (equal); Software (equal); Writing‐review & editing (equal). **Jacques Roy:** Conceptualization (equal); Methodology (equal); Writing‐review & editing (equal). **Stefan Trogisch:** Conceptualization (equal); Methodology (equal); Writing‐review & editing (equal). **Christian Wirth:** Conceptualization (equal); Methodology (equal); Writing‐review & editing (equal). **Nico Eisenhauer:** Conceptualization (lead); Funding acquisition (lead); Investigation (equal); Methodology (equal); Project administration (lead); Resources (equal); Supervision (equal); Validation (equal); Writing‐review & editing (equal).

## Figures and Tables

**FIGURE A1:**
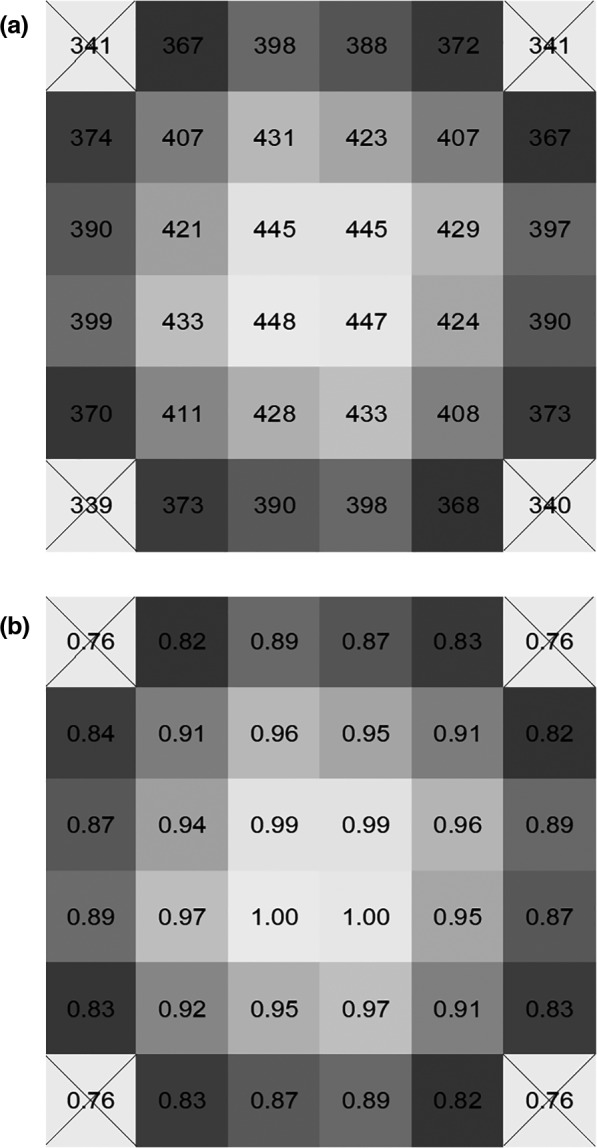
(a) Total light intensity (μmol/m^2^ s), 5 cm above the standard soil surface, at 36 locations within an EcoUnit, averaged over 24 EcoUnits, and (b) normalized deviations of light intensity at the 36 spots within an EcoUnit; normalization based on the highest measured average value of total light intensity (shown in (a)), highest value is set to 1. Five cm above the standard soil surface. Distance luminaire to backlighting layer 160 mm; outside corners are not included in calculations due to high edge effects inevitably created by the construction itself

## Data Availability

All underlying data are available from the iDiv Data Repository. https://doi.org/10.25829/idiv.3496‐8‐5695.

## APPENDIX 1

**TABLE A1:** Abiotic parameters of an EcoUnit, control, and data storage options; combined temperature/humidity sensors: *MELA FE09*, *Galltec Meß*‐ *und Regeltechnik GmbH Bondorf*, *Germany*; integrated flow meter: *FCH*‐*midi*‐*POM 97478976*, *B*.*I*.*O*.‐*TECH e*.*K*. *Vilshofen*, *Germany*; temperature/moisture sensors belowground: *SMT100*, *TRUEBNER GmbH Neustadt*, *Germany*; observation cameras: *YUC*‐*Hi82 M*, *Yudor Technology Co*, *Ltd Tao Yuan City 324*, *Taiwan*

Parameter	Controlling	User interface	Sensing
Air temperature	Via adjustable ambient temperature of hall	GUI	4 combined temperature/humidity sensors
Air humidity	Indirect only by air temperature and air flow rate	–	4 combined temperature/humidity sensors
Air flow rate	By blower speed	GUI	Manually by air velocimeter
Lighting timing	1‐h setting resolution with automatically calculated intermediate dim steps for each channel	GUI	Logging of execution confirmation only
LIGHT intensity	Nominal 1% setting resolution with internal mapping to nearest dim step	GUI	Logging of execution confirmation only
Light color mix	4 dim channels (UV, blue, red, NIR); 1 non dim channel (FIR)	GUI	Logging of execution confirmation only
Irrigation volume	50 ml setting resolution	GUI	Integrated flow meter
Irrigation timing	1‐h setting resolution	GUI	Logging of execution confirmation only
Soil temperature	By cooling at the bottom down to ~10°C with resulting temperature gradient to soil surface	GUI	Up to 12 combined temperature/moisture sensors in three levels belowground
Soil moisture	Indirect only by change of irrigation volume, soil water removal, and the manipulation of evaporation rate by air flow rate	–	Up to 12 combined temperature/moisture sensors in three levels belowground
Suction low pressure	1 kPa setting resolution with low pressure down to −60 kPa below ambient air pressure	GUI	Each suction system includes an integrated pressure sensor
Video observation	Orientation of vision and operation mode manually only	Camera's web GUI	Observation camera
Still pictures	By external script with access to video stream of running cams	Camera's web GUI + Linux shell	Observation camera

## APPENDIX 3

### Aboveground invertebrates

To test for effects of aboveground invertebrates on tritrophic interactions and nematode communities, we implemented different combinations of herbivore and predator species presence and absence. We used the pea aphid *Acyrthosiphon pisum* Harris as aboveground herbivore feeding specifically on the broad bean *Vicia faba* L. Eight mature individuals were added to each replicate of respective treatments between April 27 and May 3. We used adult beetles of the seven‐spot ladybird (*Coccinella septempunctata* Linnaeus) as specialized aphid predators which were added in two different densities (two or four individuals) on May 10 to respective treatments. In total, we tested four aboveground treatments: control (no invertebrates), herbivores only (with aphids, without ladybirds), Coccinella low (with aphids, with two individuals of *C*. *septempunctata*), and Coccinella high (with aphids, with four individuals of *C*. *septempunctata*).

## APPENDIX 4

### Details on the experimental setup

The 24 Lysimeters were filled with steam‐sterilized top soil (purchased at Bauzentrum Farys GmbH, Laucha). For sterilization, the soil was subjected to water steam at approx. 100°C for 30 min. Such sterilization leads to a heavy release of nutrients due to the death of soil organisms (Alphei & Scheu, 1993; Trevors, 1996), which is why the soil was thoroughly rinsed with tap water afterward (Jager et al., 1970). The soil was inoculated with nematode and microbial communities on February 2, 2017, marking the start of the experiment. Live soil organisms were extracted from top soil of an experimental grassland site (Jena Experiment, Roscher et al., 2004). We added four independent samples of soil wash solution (extracted from 100 g of soil each, filtered through a 125‐µm sieve) to each lysimeter on February 3. In addition, we added three independent inoculates of nematode solution between February 2 and March 10, which were previously live‐extracted from 20 g wet soil each, following the modified Baermann funnel method (Cesarz et al., 2019; for details on nematode communities in the Jena Experiment, see Eisenhauer et al., 2011; Cesarz et al., 2017). To exclude that unintended additions of nematodes might have confounded the controlled inoculation, soil samples from the sterilized soil filled into lysimeters were extracted with the same method and yielded no live nematodes. The following environmental parameters were set in the EcoUnits: light/dark cycle 16/8 h (max illumination at day, gradual change), temperature 21°C at day and 17°C at night (gradual change over the course of 3 h), irrigation of 400 ml on each lysimeter area daily at 4 am, soil temperature set to 17°C in 43.5 cm soil depth.

## APPENDIX 5

### Measurements

Numbers of aphids on each bean were counted every 7 days. For analyses, we used the peak number of all assessments during the experiment (hereafter called “maximum number of aphids”). Furthermore, we recorded the number of days beans were infested with aphids by counting live aphids on each bean individual from first discovery until last discovery; last discovery could either be the end of the experiment or the time a bean got in a bad status and was not a suitable host for aphids anymore.

All beans were harvested 49 days after their transplantation by taking 5‐cm‐diameter soil cores to a depth of 10 cm with beans in their center. The soil was sieved through a 2‐mm sieve, bean roots were extracted and both were stored at 4°C until further processing. After the removal of aphids the bean aboveground parts were dried at 45°C for 3 days and weighed. Nematodes were extracted from the previous stored soil following a modified Baermann technique with an extraction time of 48 h (Cesarz et al., 2019). Extracted nematodes were transferred to formalin (4%) and counted to obtain the total density of nematodes (given as number of individuals per 100 g dry soil). Subsequently, 100 individuals were randomly selected and identified to genus level following (Bongers & Bongers, 1998) or separated into morphospecies where not possible. Nematode species richness was calculated as (*S*−1)/ln*N*, where *S* is the number of total genera in the community, and *N* is the number of identified individuals of nematodes in the community.

## APPENDIX 6

### Soil invertebrates

The steam‐sterilized soil (Dietrich et al., 2020) got inoculated with microorganisms and nematodes (see Appendix 4). To test for interactions between the aboveground tritrophic system and belowground invertebrate presence (meso‐/macro‐fauna), we added the following soil invertebrates to one of the two lysimeters in each replicate: 15 juvenile anecic earthworms (*Lumbricus terrestris* Linnaeus, mean weight 4.4 g) and 20 individuals each of two Collembola species (*Folsomia candida* Willem, *Protaphorura armata* Tullberg). Collembola populations have been shown to develop rapidly in the experimental soil until the carrying capacity of the system is reached (Eisenhauer et al., 2011). Fifty grams of commercial grassland litter was provided as substrate to both lysimeters (bunny^®^ Frischgras‐Heu). Since hay is a natural product, its grain properties vary according to season. Typically, in Table A2 shown groups of plants are included.

**TABLE A2:** Species list of the commercial grassland litter from bunny^®^ Frischgras‐Heu provided as substrate to both lysimeters

English name	Latin name
Timothy (grass)	*Phleum pratense*
Meadow fescue	*Festuca pratensis*
Meadow foxtail	*Alopecurus pratensis*
Ryegrass	*Lolium* sp.
Red fescue	*Festuca rubra* agg.
Kentucky bluegrass	*Poa pratensis*
Bent grass	*Agrostis* sp.
Cat grass	*Dactylis glomerata*
Common dandelion	*Taraxacum officinale*
Common silverweed	*Potentilla anserina*
Mouse‐ear chickweed	*Cerastium* sp.
Yarrows	*Achillea* sp.
Ribwort plantain	*Plantago lanceolata*
White clover	*Trifolium repens*
Red clover	*Trifolium pratense*
Common bird's‐foot trefoil	*Lotus corniculatus*

## APPENDIX 7

### Plants

Nineteen 23‐day‐old seedlings each of two herbaceous (*Bellis perennis* L., *Centaurea jacea* L.) and two grass species (*Festuca pratensis Huds*., *Holcus lanatus* L.) were transplanted in regular distances of 5 cm and within monoculture quarters into each lysimeter on February 16 to mimic a simplified grassland community. In the center of each monoculture quarter, a single individual of an 8‐day‐old broad bean seedling (*Vicia faba* L., variety “Dreifach Weiße,” Bruno Nebelung GmbH) was transplanted on April 19, representing the specific host plant of aboveground herbivores. Consequently, there were four host plant individuals per lysimeter and thus eight individuals per replicate.

## APPENDIX 8

### Results of GLMM

**TABLE A3:** Effects of aboveground (AG) and belowground (BG) invertebrates and bean plant neighbor species identity as well as their interactions on five response variables using GLMM type III sum of squares analyses

Grouping variable	Response variable														
	Bean dry weight			Nematode density			Nematode sp. richness			Max. no. of aphids			Days aphid infestation		
	df	*F*	*p*	df	*F*	*p*	df	*F*	*p*	df	*F*	*p*	df	*F*	*p*
AG invertebrates	**3, 48**	**6.48**	**<.001**	3, 48	0.16	.92	3, 48	0.51	.67	**1, 24**	**8.24**	.**01**	**3, 48**	**63.19**	**<.001**
BG invertebrates	1, 48	0.26	.61	1, 48	1.01	.32	1, 48	1.99	.16	1, 24	0.22	.64	1, 48	0.20	.65
AG invertebrates*BG invertebrates	3, 48	1.37	.26	3, 48	0.84	.48	3, 48	0.12	.95	1, 24	0.25	.62	3, 48	1.25	.30
Neighbor species	**3, 48**	**5.16**	**<.01**	**3, 48**	**2.86**	.**05**	3, 48	1.62	.20	3, 24	1.42	.26	3, 48	0.36	.78
Neighbor species*AG invertebrates	9, 48	0.22	.99	9, 48	1.40	.22	**9, 48**	**2.21**	.**04**	3, 24	0.44	.73	9, 48	0.87	.56
Neighbor species*BG invertebrates	3, 48	0.30	.82	3, 48	1.25	.30	3, 48	0.62	.61	**3, 24**	**5.82**	**<.01**	3, 48	1.58	.21
Neighbor species*AG invertebrates*BG invertebrates	9, 48	0.92	.52	9, 48	0.76	.65	9, 48	0.86	.56	3, 24	0.24	.87	9, 48	0.30	.97

Note

Significant effects (*p* < .05) are indicated in bold font.

Abbreviations: max. no. of aphids, maximum number of aphids; sp. Richness, species richness.
